# Do You Mean What You Say?

**Published:** 1876-03

**Authors:** 


### *DO YOU MEAN WHAT YOU SAY?*

A reader of the JOURNAL asks us this question with reference to some encomiums which we, from time to time, have passed upon certain articles offered for sale. Once for all let us say we *do* mean precisely what we say about men and things. We do not publish "puffs," whatever the inducements offered. If we know a thing is good, we are very apt to say so; and it is not uncommon for us to say it more than once. But we mean it, every time. If we know of a so-called "patent medicine" which is useful in its way, we say so, without regard to results. The most eloquent preacher we ever listened to once remarked that if he were sick and knew of a remedy which would relieve him, he would have that remedy no matter what schools of medicine might oppose its use. This is good sense. We scour the country for good and useful things, and when we find such we never hesitate to ask our readers to share with us the benefits. That they appreciate this is abundantly shown. Last summer we received letters almost without number from parents expressing deep gratitude for the benefits derived by little sufferers from the use of the sugary pellets called "Van Deren's Remedy for Cholera Infantum." Some of our medical brethren said we were advocating a homœopathic medicine contrary to the ethics of the various medical societies to which we belong. Such charges do not trouble us. We know that these pellets SAVE LIFE, and that is enough for us. So of the Nelaton Pile Suppository. We indorse it because it is a splendid remedy for a painful and sometimes dangerous complaint. We would not be without these two remedies in our practice for much money, because we know of nothing which would take their place.

Let it be known, then, that the utterances in HALL'S JOURNAL OF HEALTH are our own, and that what we enunciate as a fact, is the honest truth; and what we give as our judgment is our honest judgment, resulting from careful and conscientious investigation. We write in the interest of our readers, and in the interest of nobody else. There is not a valuable thing in our line in the world which we will not commend to our readers if we are able first to satisfy ourselves of its superior excellence.

